# Comparison of cycle-threshold-values between two commercial SARS-CoV-2 PCR assays

**DOI:** 10.17179/excli2023-5981

**Published:** 2023-04-04

**Authors:** Simon Michaelis, Christopher Schneider, Wolfgang J. Schnedl, Andreas Baranyi, Dietmar Enko

**Affiliations:** 1 Institute of Clinical Chemistry and Laboratory Medicine, General Hospital Hochsteiermark, Vordernberger Straße 42, 8700 Leoben, Austria; 2 Practice for General Internal Medicine, Dr.-Theodor-Körner-Straße 19b, 8600 Bruck/Mur, Austria; 3 Department of Psychiatry and Psychotherapeutic Medicine, Medical University of Graz, Auenbruggerplatz 31, 8036 Graz, Austria; 4 Clinical Institute of Medical and Chemical Laboratory Diagnostics, Medical University of Graz, Auenbruggerplatz 15, 8036 Graz, Austria

## ⁯

The cornerstone in the diagnosis of SARS-CoV-2 is the detection of the viral RNA by PCR methods. These assays are approved as qualitative tests, hence they are generally not intended for quantification purposes. However, during the pandemic the need for a quantitative measure of SARS-CoV-2 emerged. This demand was particularly urging for the clinical management of infected patients in questions of isolation and the assessment of infectiousness (Rao et al. 2020[8]). Thus, the cycle-threshold (Ct)-value, which is reported by the PCR assays for each tested gene target, was introduced in order to enable clinical decisions (Rao et al. 2020[8]). There is an ongoing debate, if the use of Ct-values in the clinical context is appropriate (Stokes et al., 2021[10]). Among others, the lack of standardization and PCR inter-assay comparability are arguments against the usage of Ct-values (Stokes et al., 2021[10]). In this study we aimed to compare the Ct-values obtained from two commercially available SARS-CoV-2 PCR test systems used in our laboratory.

Overall, 154 specimens from individuals tested positive for SARS-CoV-2 in our laboratory with the GeneXpert Xpert SARS-CoV-2 assay (Cepheid, Sunnyvale, CA, USA) were collected between January and April 2022. They were frozen at -80 °C. After thawing they were used for parallel processing on the GeneXpert Xpert SARS-CoV-2 and the NeuMoDx SARS-CoV-2 assay (Qiagen, Waltham, MA, USA). The Cepheid Xpert SARS-CoV-2 test is an RT-PCR assay utilizing the nucleocapsid 2 (N2) and the envelope (E) genes as targets on the SARS-CoV-2 genome. The NeuMoDx SARS-CoV-2 test uses the nucleocapsid (N) gene and the non-structural protein (Nsp2) gene as PCR targets.

The Ct-values provided by the GeneXpert were generally higher compared to those from the NeuMoDx. Within the GeneXpert platform, the Ct-values for the N2-target were higher than for the E-target. The NeuMoDX showed higher Ct-values for the Nsp2-target compared to the N-target. Pairwise comparisons showed statistically significant differences between all tested gene targets (Wilcoxon test, all *P*-values < 0.01). The comparison between the two platforms showed the highest difference between the N2 gene target on the GeneXpert and the N gene target on the NeuMoDx (mean absolute difference 3.6, 95 % limits of agreement 1.0 - 6.5). The lowest difference was observed between the E gene target on the GeneXpert and the Nsp gene target on the NeuMoDx (mean absolute difference 1.1, 95 % limits of agreement -2.3 - 4.5). The intra-assay differences for the various gene targets were both statistically significant with a higher magnitude in the GeneXpert assay (N2 target vs. E target: mean absolute difference 2.0, 95 % limits of agreement 0.4 - 3.6) compared to the NeuMoDx (N target vs. Nsp target: mean absolute difference -0.6, 95 % limits of agreement -1.6 - 0.3). Statistically significant correlations were observed between all different gene targets (all *P*-values <0.01) The highest percentage of measurements with a Ct-value of >30 showed the PCR targeting the N2 gene on the GeneXpert (25.3 %), while the PCR of the N gene on the NeuMoDx was lowest with 10.4 % of the results over a Ct-value of 30. In seven specimens (4.5 % of all specimens) the Ct-values from the GeneXpert were >30 in both gene targets, whereas the Ct-values obtained from the NeuMoDx were <30 in both gene targets. There was no case with the opposite constellation. 

Soon after the implementation of PCR assays for the detection of SARS-CoV-2, the Ct-value began to gain attention (Rao et al., 2020[8]). An association between low Ct-values with severe disease, higher mortality and the presence of unfavorable biomarkers was reported (Rao et al., 2020[8]). The Ct-value has been particularly used for the clinical decision, whether a positively tested individual should be de-isolated or not (Aranha et al., 2021[2]). As a cut-off for the decision-making regarding isolation, various Ct-values were proposed (Al Bayat et al., 2021[1]; Aranha et al., 2021[2]). In general, these Ct-values are ranging around 30. From the clinician's viewpoint, this approach has valuable advantages. If there is a clear cut-off, algorithms for the patient isolation and discharge procedures can be established and applied straightforwardly. However, the use of the Ct-values as the basis for clinical decisions is controversial. The Ct-value is generally used as a proxy for viral load. Therefore, to ensure comparability, standardized patient specimens would be needed. In the case of nasopharyngeal swabs, this is not achieved easily. The quantity of viral RNA in the clinical specimen depends on numerous variables including the site of the swab collection, the swabbing technique, the used transport media and volume as well as the virus inactivation procedures prior to testing (Rhoads and Pinsky, 2021[9]). Moreover, SARS-CoV-2-RNA extraction and amplification procedures vary considerably. Manufacturers of PCR systems use different PCR protocols and reagents, especially depending on the respective gene target (Poon and Wen-Sim Tee, 2021[6]). In the case a PCR system uses two gene targets (dual-target PCR) or three gene targets (triple-target PCR), completely different Ct-values may be obtained. The commercially available PCR tests for SARS-CoV-2 are solely approved for the qualitative analysis of the viral RNA. Since Ct-values are inherent measures to the specific PCR protocols, there is no standardization or even calibration in place. Comparisons between various PCR systems have demonstrated considerable differences in the reported Ct-values (Buchta et al., 2021[4]; Broder et al., 2020[3]; Moran et al., 2020[5]; Raju et al., 2021[7]). In a comparison of six SARS-CoV-2 PCR platforms, Raju et al. (2021[7]) observed diverging Ct-values between the test systems as well as between the different gene targets within the respective test systems. Buchta et al. (2021[4]) reported the analysis of an external quality assessment program for the molecular detection of SARS-CoV-2. Systematic deviations between the test systems and the respective gene targets were observed (Buchta et al., 2021[4]). Moran et al. (2020[5]) compared the Ct-values for the E gene target between the GeneXpert and another PCR system. The values were lower when using the GeneXpert (Moran et al., 2020[5]). Values from the GeneXpert for the E gene were reported to be lower than those for the N2 gene (Buchta et al., 2021[4]). This is in accordance with our data. We observed higher Ct-values for the N2 gene compared to those for the E gene. Furthermore, we demonstrated a statistically significant difference of the Ct-values between the GeneXpert and the NeuMoDx. To our knowledge this is the first comparison of Ct-values for SARS-CoV-2 from the NeuMoDx with another PCR system. Overall, the NeuMoDx showed lower Ct-values compared to the GeneXpert. In seven specimens (4.5 %) the Ct-values obtained from the GeneXpert were >30, while at least one Ct-value from the NeuMoDx was <30. Hence, if applying a Ct-value of 30 as the cut-off value for assumed infectivity or isolation, the consequences inferred from the test results would be different. Thus, Ct-values as a quantitative measure with a fixed cut-off are to be used with caution. Patients might be released from isolation when using one PCR assay, while they might need to stay in isolation when using a different one.

In conclusion, Ct-values for SARS-CoV-2 are merely imperfect representations of a viral load. Ct-values between PCR tests can vary significantly. For an actual quantification of the viral RNA, standards with known RNA concentrations in order to generate a calibration curve should be implemented. Furthermore, an international standardization for comparisons between PCR tests would be of advantage. 

## Conflict of interest

The authors declare no conflict of interest.
